# Phenolic Composition of Grape Stems from Different Spanish Varieties and Vintages

**DOI:** 10.3390/biom11081221

**Published:** 2021-08-16

**Authors:** Irene Esparza, José Antonio Moler, Maite Arteta, Nerea Jiménez-Moreno, Carmen Ancín-Azpilicueta

**Affiliations:** 1 Department of Sciences, Universidad Pública de Navarra, Campus Arrosadía s/n, 31006 Pamplona, Spain; irene.esparza@unavarra.es (I.E.); maiteac@hotmail.es (M.A.); 2 Institute for Advanced Materials (INAMAT2), Universidad Pública de Navarra, 31006 Pamplona, Spain; 3 Department of Statistics and Operational Research, Universidad Pública de Navarra, Campus Arrosadía s/n, 31006 Pamplona, Spain; jmoler@unavarra.es

**Keywords:** grape stem, variety, vintage, phenolic composition, antioxidant activity

## Abstract

Grape stems are a by-product from the wine industry that has been underused to date despite having great potential for the agro-food and cosmetic industries. The aim of the present work was to characterize grape stem extracts obtained from different grape varieties from two vintages (2016 and 2018). Both spectrophotometric and chromatographic methods were used for sample characterization. The results showed that there exist significant differences in antioxidant activity, total phenolic content (TPC) and total flavonoid content (TF) among grape stems from different varieties in each vintage and from different vintage for the same variety. Catechin was the most abundant phenolic compound in all extracts from both vintages. In general, Mazuelo presented higher concentration values of the different phenolic compounds than Garnacha and Tempranillo. It was observed than extreme temperatures and accumulated precipitations, which were higher in the 2016 vintage, had an impact on the polyphenol synthesis. Therefore, grape stems from the 2018 vintage presented higher TPC and TF values than their counterparts from the 2016 vintage. In addition, the statistical analysis revealed that the influence of environmental factor such as light, temperature and precipitations have different impact on the synthesis of polyphenols depending on the family of the specific compound.

## 1. Introduction

The agricultural sector and the agro-food industry generate a large quantity of problematic residues because of their high humidity and also their high content of organic matter. One of those industries corresponds to the wine industry, that produces high levels of waste, which is a major problem and concern from both economic and ecological point of view [1]. The main by-products of this industry include grape stems, skins and seeds, that involves 20% of the weight of the processed grapes [2]. Currently there exist innovative applications for the use of these residues, including the use of grape skins for the production of infusions [3], food supplements [4] or as additives in the winemaking process itself [5]. Among them, grape stem are the least studied by-products despite representing a large amount of annual waste [6]. Usually, grape stems are used as organic fertilizer, as feed for ruminants [7] or valorized through the production of ethanol and paper pulp [8]. However, in recent years, several studies have demonstrated that these by-products contain high concentrations of polyphenols, cellulose, hemicellulose and lignin [9,10,11], which is causing a growing interest in their use as bioactive phytochemicals [12,13]. For instance, Leal et al. [14] found that stem extracts present antimicrobial activity, with high efficacy against ulcers produced by Gram-positive bacteria in foot wounds. In addition, they observed that these extracts have an anti-inflammatory capacity by inhibiting the production of nitric oxide by lipopolysaccharide-stimulated macrophages, and anti-aging effect by inhibiting the anti-tyrosinase and anti-elastase activities. Grape stem extracts have also been demonstrated to be a good therapeutic agent, as they possess antibacterial activity against a wide variety of pathogens, including *Listeria monocytogenes, Staphylococcus aureus, Enterococcus faecalis, Escherichia coli, Pseudomonas aeruginosa* and *Salmonella enterica* [15,16]. Other authors also found that the use of these by-products in food industry, in cosmetics and in products with pharmacological activity is interesting since they have potential antioxidant, antimicrobial, anti-inflammatory and anticancer activity, among others [17,18,19,20].

Several authors have demonstrated that the antioxidant activity of grape stem extracts is correlated with their phenolic composition [7,12,16,21,22]. There exist a large variety of phenolic compounds belonging to different families identified in grape stems. Among them, caftaric and gallic acids are the most representative phenolic acids found in grape stems, *trans*-resveratrol and ε-viniferin the two main stilbenes, quercetin derivatives the main flavonols, catechin and epicatechin the most abundant flavanols, and malvidin-3-glucoside and other malvidin derivatives are the anthocyanins most frequently identified in those by-products [9]. Despite this, Teixeira et al. [23] found that the composition and concentration of polyphenols on grape stems depend on the grape variety, ripeness, climatic and geographical growing conditions and cultivation practices. However, the magnitude of the influence of each parameter in the phenolic composition of grape stem it is not well known. There exist research works that studied the influence of the grape variety on the phenolic composition of grape stem extracts [21,24,25], but there are few studies that afford the influence of a relevant factor such as the vintage in the polyphenolic composition of grape stems. Recently, Blackford et al. [9] performed a compilation of the phenolic composition of different grape stems reported in the literature by different authors. In this work it is confirmed that there exist differences in the phenolic composition and antioxidant capacity among stems from different varieties and vintages. However, it is very difficult to compare the values obtained from different studies, since the extraction protocols are different, and, also, data are presented in different units. For this reason, the main objective of the present work is to know how the grape variety and the vintage can affect the phenolic composition of grape stem extracts from six varieties (Garnacha, Tempranillo, Mazuelo, Graciano, Chardonnay and Cabernet Sauvignon), all of them from the same geographical region, and three of them coming from two different vintages (Garnacha, Mazuelo and Tempranillo). The use of samples from the same geographical region will guarantee that the influence of soil, culture practices and climatic conditions will be very similar in all cases, so the use of the same extraction and analysis protocols will allow obtaining reliable information about the influence of the variety and vintage on the phenolic composition of grape stem extracts.

## 2. Materials and Methods

### 2.1. Samples

Grape stem samples of different varieties were collected at the Navarra Viticulture and Oenological Research Station (EVENA), located in Olite (northern Spain). In the 2016 vintage, samples from Graciano, Mazuelo, Tempranillo and Garnacha varieties were collected, and in the 2018 vintage, the grape stem collected were from Garnacha, Mazuelo, Tempranillo, Chardonnay and Cabernet Sauvignon varieties. Each grape variety was harvested at its optimum degree of ripeness. At the 2016 harvest, the degree of maturation (expressed as °Brix) of the Garnacha grape was 24.5°, the Tempranillo grape presented 23.8°, 23° in the Mazuelo grape and the Graciano variety presented 21.6°. At the 2018 harvest, the degree of grape maturation was 21.4° in the case of Chardonnay variety, 23.7° in Cabernet Sauvignon grapes, 25.9° in Garnacha grapes, 24.8° in Tempranillo grapes and 23° in grapes from Mazuelo variety. After harvest, grape stems were dried at 25 °C in a stove (Ing Climas, Barcelona, Spain) until constant weigh. Then, dried grape stems were milled in a coffee grinder (Moulinex, Ecully, France) and sieved with a sieve of 300 µm. Later, the dried and milled grape stems were incubated in a 50% *v/v* ethanol/water solution (solid/liquid ratio 1:100 *w*/*v*) for 24 h at 40 °C. Finally, the resulting extracts were centrifuged (8000 rpm, 15 min), filtered and freeze-dried. The extraction process was conducted in triplicate for each sample of grape stems. 

### 2.2. Determination of the Antioxidant Capacity, Total Phenolic and Total Flavonoid Content of the Grape Stem Extracts

The antioxidant capacity of the different extracts was measured by the ABTS, DPPH and FRAP methods. The ABTS (2,2′-azinobis(3-ethylbenzothiazoline-6-sulphonic acid)) method used is based on the method described by Re et al. [26]. Briefly, the ABTS*^●+^* radical was obtained after 16 h of incubation in darkness of a solution of ABTS 7 mM and potassium persulphate, 2.45 mM. The calibration curve was prepared from a 5 mM Trolox solution, and the concentration range was from 0.05 to 1.2 mM. Prior to absorbance measurement, 30 µL of Trolox standard or grape stem extract (previously dissolved by triplicate in methanol at concentration from 0.25 to 1.25 mg/mL) were mixed with 2.97 mL o the ABTS*^●+^* radical solution. After 30 min in darkness., samples were finally measured at 734 nm with a UV/Vis spectrometer (Jenway 7315, Staffrodshire, UK). 

The DPPH (2,2-diphenyl-1-pycrilhydracyl) assay used in this work was based on the method previously described by Brand-Williams et al. [27]. Firstly, a 0.24 mg/mL of DPPH solution was prepared in ethanol. 10 mL of this solution were then diluted in methanol until obtaining an absorbance of 1.1 ± 0.1 at 515 nm. For the calibration curve, Trolox was used as standard with a concentration range of 0.05 to 0.56 mM. For analysis, 150 µL of the Trolox standard solution or sample (grape stem extract previously diluted in methanol at a concentration of 0.5 mg/mL) were mixed with 2.85 mL of the DPPH solution. After 30 min in darkness, the absorbance was measured at 517 nm.

The antioxidant activity of the different extracts was also measured by the FRAP method proposed by Benzie and Strain [28]. This method is based on the reduction in acid medium of the complex Fe^3+^-TPZ to the ferrous form in presence of antioxidants. Known concentrations of Trolox, in the range of 0.05 to 0.5 mM were used for preparing the calibration curve. For sample measurement, 150 µL of the Trolox standard solution or grape stem extract (previously dissolved in methanol at a concentration of 0.5 mg/mL) were mixed with 2.85 mL of FRAP reagent (acetate buffer 300 mM: 2,4,6-tris(2-pyridyl)-s-triazine 9.99 mM: FeCl_3_·6H_2_O 20 mM, 10:1:1), and left for 30 min in darkness. Finally, absorbance was measured at 595 nm.

For the quantification of total polyphenol content (TPC), the Folin–Ciocalteu method described by Singleton et al. [29] was used. Calibration curve was prepared from gallic acid in concentrations ranging from 0.2 to 4.6 mM. For sample measurement, 0.1 mL of the gallic acid standard or sample (grape stem extract dissolved in methanol at a concentration of 2.5–5 mg/mL) were mixed with 0.5 L of the Folin–Ciocalteu regent, 7.9 mL of deionized water and 1.5 mL of Na_2_CO_3_ (20% w/w). After 2 h in darkness, the absorbance of the resulting solutions was measured at 765 nm.

Finally, the total flavonoid content (TF) was assessed through a colorimetric method based on the reaction of flavonoids with aluminum chloride in acetic acid described by Chandra et al. [30]. For the calibration curve, a quercetin commercial standard was used in concentrations between 3 and 30 µg/mL. For sample analysis, 1.5 mL of standard or grape stem extract (dissolved in methanol at a concentration of 5 mg/mL) were mixed with 1.5 mL of a 2% AlCl_3_ solution (prepared in 5% acetic acid), and the resulting solutions were left for 30 min in darkness. Finally, absorbance was measured at 420 nm.

For each of the three extracts obtained from each grape stem sample, two different processed samples were prepared and each of them was analyzed once by all the spectrophotometric methods. The linear correlation coefficient obtained for the calibration curve was R^2^ > 0.998 in all cases. The results of antioxidant capacity were expressed as mmol Trolox/g of freeze-dried extract, the results of TPC were expressed as mg gallic acid/g extract, and the results of TF as mg quercetin/g extract.

### 2.3. Identification and Quantification of Phenolic Compounds by HPLC-DAD

The phenolic composition of the extracts was determined by HPLC by using a high-performance liquid chromatograph equipped with two 510 pumps, a 717 Plus autosampler, and a 996 photodiode array detector (Waters Div., Milford, MA, USA). The column used for the analysis was a reversed-phase column (Zorbax Eclipse Plus C18, 250 × 4.6 mm, particle size 5 µm, Agilent, Santa Clara, CA, USA) at 30 °C. The control of the instrument and data processing were conducted by using the Empower 2.0 software. For the analysis of the extracts, between 12.0 ± 0.1 and 38.0 ± 0.1 mg of each sample were weighted and dissolved in 350 µL of methanol with the aid of an ultrasonic bath (JP Selecta, Barcelona, Spain). Samples were prepared in triplicate and analyzed once. 

The chromatographic separation of the different phenolic compounds was conducted according to a modified method of Barros et al. [12]. Two mobile phases were used: A (water: formic acid 85%, 99:1 *v*/*v*) and B (acetonitrile: formic acid 85%, 99:1 *v*/*v*), and the gradient was the following (t in min: % A): (0; 95%), (15, 85%), (22, 80%), (25, 80%), (35, 70%), (45, 50%), (50, 5%), (55, 95%) and (60, 95%). The flow rate was 1 mL/min and the injection volume was 10 µL. All solvents used for the analysis were of HPLC quality (Scharlab, Barcelona, Spain).

The identification of each phenolic compound was conducted by the coincidence of both the UV-Visible characteristic spectrum and the retention time of its corresponding standard. The identified phenolic compounds were: gallic acid, catechin, malvidin-3-glucoside, caftaric acid, ellagic acid, procyanidin B1, quercetin, a quercetin-3-derivative (quantified and expressed as quercetin-3-glucoside), *trans*-resveratrol, ε-viniferin and an unknown anthocyanin. Quantification was carried out using calibration curves for each analyzed compound with the exception of the unknown anthocyanin that was quantified and expressed as malvidin-3-glucoside. The correlation coefficients of the calibration curves used were R^2^ > 0.99 in all cases, and the coefficients of variation of the response factors (calculated as the relation between the response and the concentration of each standard) were lower than 13% in all cases. The detection limit (LLOD) was calculated by the following expression: LLOD = 3.3·σ/S, were S is the slope of the calibration curve and σ is the standard deviation of the regression line. The quantification limit (LLOQ) was the lowest concentration included in the calibration range. Table 1 shows the calibration parameters for each specific compound. 

### 2.4. Statistical Analysis

The experiment contemplates a nested structure that groups data as follows. For each wine variety specimen, a set of three extractions were obtained and, then, depending on the analysis, two or three measurements were obtained for each variable of interest. In order to consider these hierarchical levels of grouped data in the estimation of the variance, a hierarchical linear model is adjusted to these data. Data processing has been carried out with R [31] and post-hoc analysis to stablish differences among varieties for the hierarchical model has been performed with the “lsmeans” package [32].

## 3. Results and Discussion

### 3.1. Antioxidant Capacity, TF and TPC

Table 2 shows the results of the spectrophotometric analysis performed on all samples. The antioxidant activity and TPC values were in the same order than those obtained by other authors [9,14,24]. As it can be seen, there exist significant differences in antioxidant activity, TPC and TF content among grape stems from different varieties in each vintage. However, such differences are not maintained from one vintage to another. In the 2016 vintage, the Garnacha stem extract presented the lowest levels of TPC, TF and antioxidant capacity, while the rest of extracts (from Mazuelo, Tempranillo and Graciano varieties) did not present significant differences in TPC, TF and antioxidant capacity (measured by ABTS) among them. In the 2018 vintage, the extract from Garnacha stem also presented significant lower levels in all the parameters than those obtained for extracts from Mazuelo and Tempranillo varieties. However, in this case, Mazuelo and Tempranillo extracts did show significant differences between them. In that vintage (2018) the Mazuelo stem extract was the one with the highest values of TPC, TF and antioxidant activity. The two new extracts analyzed in 2018 (from Chardonnay and Cabernet Sauvignon varieties) presented, together with the extract from Garnacha, the lowest levels of antioxidant capacity, TPC and TF. Other authors have also found differences on the antioxidant activity and phenolic content among stems of different grape varieties. González-Centeno et al. [24] studied the antioxidant potential of different grape stem varieties, and found that stems from Callet, Syrah, Premsal Blanc, Parellada and Manto Negro varieties yielded the highest total phenolic and total proanthocyanidin contents and showed the greatest antioxidant capacities, whereas Chardonnay and Merlot stems presented the lowest values. Varieties differed significantly (*p* < 0.05) with regard to both the phenolic composition and antioxidant capacity of their stems. However, no significant differences (*p* > 0.05) were observed when stems from red and white varieties were considered separately. Moreover, Leal et al. [14] evaluated the antioxidant capacity of grape stem from different Portuguese varieties (Tinta Roriz, Touriga Nacional, Castelão, Syrah, Arinto and Fernão Pires) and observed significant differences among varieties from the same vintage (2018). When comparing the results obtained in the present study with those obtained by Leal et al. [14], we observe that they found antioxidant capacity values between 0.35 ± 0.00 and 0.84 ± 0.06 mmol Trolox/g in their samples, and these values are similar than those obtained in the present work. 

In their exhaustive review on grape stem composition, Blackford et al. [9] mentioned that there is no standardized method in the literature to characterize the antioxidant potential of grape stem extracts, but ABTS, DPPH and FRAP are the most used methods. In the present study, the antioxidant activity values obtained by the ABTS assay were higher than those obtained by the other two antioxidant methods. This is in agreement with previous studies conducted for this type of samples [12,19,20]. Despite ABTS and DPPH share the same mechanism of action, the radical site of the DPPH molecule is located inside a reaction cage formed by two phenyl rings orthogonal to each other, and the pycril ring angled at about 30° with two nitro groups oriented above and below the radical site [33]. Therefore, steric accessibility is a limiting factor of the DPPH reaction, which could explain the lower values observed when using this method compared to those obtained by using the ABTS assay. 

Furthermore, it should be noted that results obtained by ABTS in the present work showed similar statistical differences among varieties than those of TPC and TF, but it was not the case for DPPH and FRAP results. In view of these results, and given the fact that most of the antioxidant capacity of the grape stem extracts could be attributed to their polyphenolic composition [7,12,16,21,22], it could be considered that among the three methods used in this work, ABTS seems to be the most adequate for estimating the antioxidant capacity of grape stems.

When comparing results from different vintages, it was found that, in general, extracts from all varieties from the 2018 vintage presented significant higher values than their counterpart from the 2016 vintage (see Figure S1 in Supplementary Material). Considering that in all cases samples were obtained from the same vineyard and subjected to the same viticultural practices, it could be stated that the main cause of the differences among them from one vintage to another is the weather conditions. According to Teixeira et al. [34], the factors with the greatest influence in phenolic synthesis are light/radiation and temperature, as well as water and nutritional status. Therefore, in order to understand the influence of such conditions on the composition of grape stem extracts, the daily data registered by the Olite weather station (max. temperature, min. temperature, average temperature, max. relative humidity, min. relative humidity, accumulated precipitation and radiation flux expressed in W/m^2^) over the period of grape development, veraison and ripeness process (June to October) were consulted. In order to compare both vintages, the monthly average of the different parameters were calculated. Surprisingly, no clear differences were found in average temperatures and relative humidity (maximum, minimum and average) between the two years. However, it is described that the synthesis of some polyphenols such as anthocyanins decreases when temperature conditions are extreme (too high or too low) [35]. For these reasons, instead of averages, we also determined the number of days within the mentioned period of time with maximum temperatures equal or higher than 35 °C, and minimum temperatures equal or lower than 10 °C. From June to October of 2016, there were 11 days in which maximum temperatures ≥ 35 °C were reached, and 16 days in which the minimum temperatures descended to values ≤ 10 °C. However, in 2018 the number of days were 7 and 9, respectively, which means that in 2016 there was a larger number of days with extreme conditions than in 2018. On the other hand, the sum of accumulated precipitations in this period of time were 58.5 L/m^2^ and 52.2 L/m^2^ for years 2016 and 2018, respectively, and the radiation flux were 38,513.8 W/m^2^ and 40,724.3 W/m^2^ for years 2016 and 2018, respectively. In this context, Ferrer-Gallego et al. [36] studied the influence of climatic conditions on the phenolic composition of *Vitis vinifera* L. cv. Graciano, concluding that anthocyanin and flavanol concentrations in grapes presented a direct relationship with accumulated solar radiation and an inverse relationship with the accumulated rainfall. These conclusions are in agreement with our results, as all the samples from the 2018 vintage presented higher TPC and TF values than their counterparts from the 2016 vintage, being the accumulated rainfall lower and the accumulated solar radiation higher in 2018. On the other hand, it is well known that biosynthetic pathways of different phenolic compounds involve, firstly, the deamination of phenylalanine and its transformation in cinnamic acid by the phenylalanine ammonia lyase (PAL) enzyme [34], and the activity of this enzyme is enforced by solar radiation and inhibited in absence of light [37]. Therefore, the increase in the radiation flux observed in 2018 with respect to 2016 may have favored a higher activity of this enzyme in the biosynthesis of polyphenols, what is also in agreement with the results obtained in the present work. 

### 3.2. Phenolic Compounds

Table 3 shows the phenolic compounds identified and quantified in grape stems from the different varieties from the 2016 and 2018 vintages. These data are also summarized through an inferential analysis of the phenolic composition in Figures S2 and S3 (see Supplementary Material). As it can be seen, catechin was the most abundant phenolic compound in all extracts from both vintages, with a concentration range between 0.95 and 3.50 mg/g. This result is in agreement with other authors [10,23,38,39] who also identified catechin as one of the main polyphenolic compounds in grape stem extracts, and described similar concentration ranges than those obtained in the present study. Among the phenolic acids identified and quantified in the present study, gallic acid was the most abundant in all varieties and vintages, with concentration values from 0.12 to 1.29 mg/g. These results are consistent with other authors [10,18] who also found that gallic acid is one of the most abundant hydroxybenzoic acids in grape stem extracts. 

In the 2016 vintage, grape stem extracts of the studied varieties showed different phenolic profiles. Moreover, the concentration of individual compounds showed significant differences among them, with the exception of ellagic acid, which presented similar concentration levels in samples from different varieties. Regarding samples from each variety, Graciano stem extract presented the highest values in anthocyanins, procyanidin B1, catechin and *trans*-resveratrol, while Mazuelo stem extract was richer in gallic acid, ε-viniferin, quercetin and quercetin-3-derivative. Garnacha samples presented the lowest values of all the phenolic compounds analyzed, especially of the unknown anthocyanin, quercetin-3-derivative and caftaric acid. Finally, extracts from Tempranillo stems presented similar concentration than Garnacha extracts in all components with the exception of anthocyanins, caftaric acid, quercetin and its derivative, which presented significantly higher values.

In 2018, stem extracts from Mazuelo variety presented the highest values of procyanidin B1, resveratrol, gallic acid, quercetin and quercetin-3-derivative, and extracts from Tempranillo stems were the richest in anthocyanins. It should be point out that stem extracts from Chardonnay (the only white variety analyzed in this work) showed the lowest concentrations of all the components with the exception of stilbenes (ε-viniferin and *trans*-resveratrol). This is in line with Gouvinhas et al. [10] who compared several research works and concluded that, despite some exceptions, grape stems from red varieties have significantly higher values of phenolic compounds than stems from white varieties.

When comparing the phenolic composition of grape stem extracts of the same variety but from two different vintages, most of the polyphenols showed significantly higher concentrations in extracts from the 2018 vintage than from the 2016 vintage (see Table 3). In the case of Garnacha stem extracts, only catechin and the unknown anthocyanin showed similar values in both vintages, while in the case of Tempranillo stem extracts, catechin, ε-viniferin, procyanidin B1 and *trans*-resveratrol were the phenolic compounds that kept similar values from one vintage to another (see Figure S4 in Supplementary Material). Finally, Mazuelo stems showed higher values of all the phenolic compounds in 2018 than in 2016 with the exception of ε-viniferin that presented lower values in 2018 than in 2016. Nevertheless, and despite the fact that most of the phenolic compounds increased in concentration from one vintage to another, it can be observed that not all of them presented the same enhancement.

**Figure 1:**
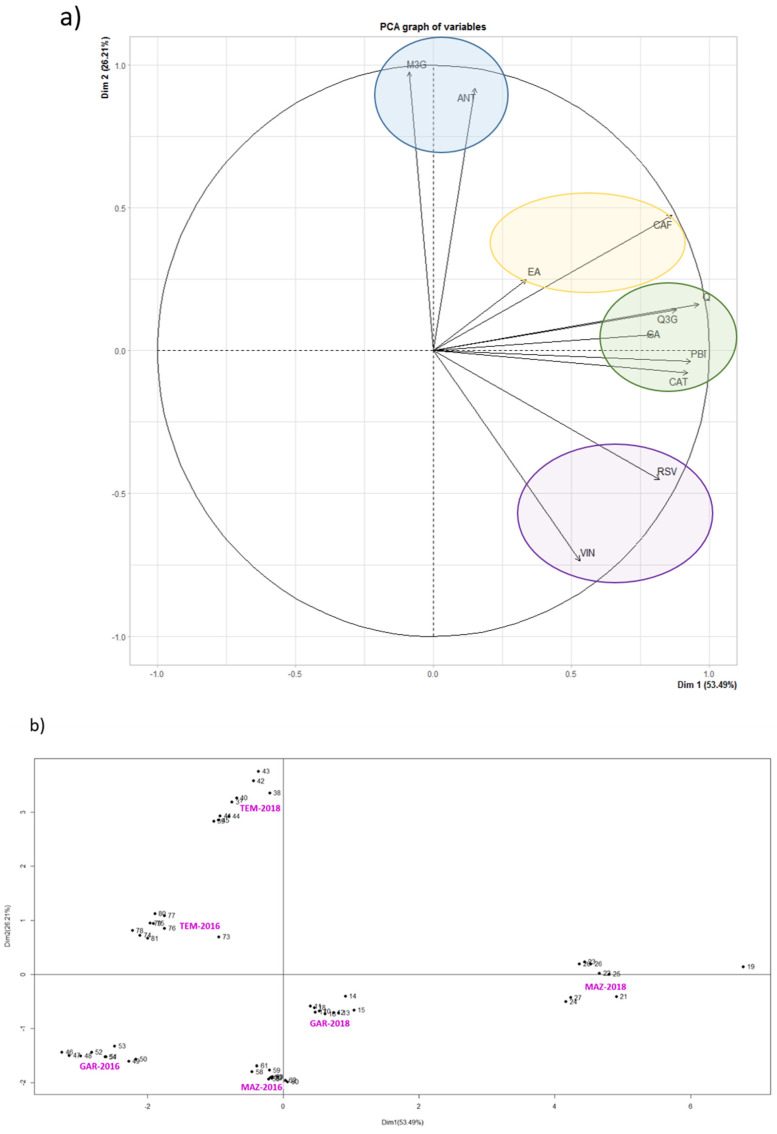
Principal component analysis (PCA) of phenolic compounds of grape stem extracts from Mazuelo, Garnacha and Tempranillo varieties from vintages 2016 and 2018. (**a**) Variables graph: GA (gallic acid); CAT (catechin); Q (quercetin); Q3G (quercetin-3-derivative); M3G (malvidin-3-glucoside); ANT (unknown anthocyanin); RSV (trans-resveratrol); VIN (ε-viniferin); CAF (caftaric acid); EA (elagic acid); PB1 (procyanidin B1); (**b**) Individuals factor map. Individuals (Mazuelo 2016 and Mazuelo 2018; Graciano 2016 and Graciano 2018; Tempranillo 2016 and Tempranillo 2018) are presented in pink color.

In order to better understand the behavior of each phenolic compound, results of grape stem extracts from Mazuelo, Garnacha and Tempranillo varieties were subjected to a principal component analysis (PCA) (Figure 1). As it can be seen in Figure 1a, PCA grouped the variables under study into four different clusters. The first one is composed by both anthocyanins quantified in this work. As we move along the plane in clockwise direction, the following cluster is composed by ellagic and caftaric acid, the third cluster by quercetin and its derivative, gallic acid, catechin and procyanidin B1, and the fourth cluster is defined by the two stilbenes, ε-viniferin and *trans*-resveratrol. In both Figure 1a,b, the OX axis is explained by the phenolic composition, so it only informs about the increase of the concentration of phenolic compounds from 2016 to 2018. In Figure 1b (individuals chart) it can be seen that grape stem extracts from vintage 2018 are shifted to the right of the OX axis, what indicates that those samples presented higher concentrations of phenolic compounds than their counterparts of the 2016 vintage.

Nevertheless, and despite that the concentration of all variables increased from 2016 to 2018, the phenolic compounds contained in the third cluster were the most affected from the “vintage” factor. Therefore, gallic acid, quercetin, quercetin-3-derivative, catechin and procyanidin B1 presented the highest increase in concentration from 2016 and 2018, and this effect is particularly remarkable in Mazuelo stem extracts (Figure 1b). 

On the other hand, it can be observed that both malvidin-3-glucoside and the unknown anthocyanin, which correspond to the first PCA cluster, are the compounds that best reflect the position of Tempranillo stem extracts in Figure 1b. It seems that grape variety has a more decisive influence on the concentration of these compounds in grape stems than vintage. Similar conclusions can be obtained for both stilbenes, as the variation in concentration levels of both *trans*-resveratrol and ε-viniferin is higher among varieties than between vintages. In the case of stilbenes, it also seems that these compounds are the phenolics least affected by weather conditions during grape development and veraison, since there has hardly been variation in their concentration from one year to another in any of the varieties studies in comparison with the rest of phenolic compounds analyzed. 

In view of all these results, it could be concluded that the biosynthesis and accumulation of anthocyanins in grape stems between vintages is not correlated with either the behavior observed for the flavan-3-ols (catechin), proanthocyanidins (procyanidin B1), flavonols (quercetin and its derivative) and hydroxybenzoic acid (gallic acid), or the stilbenes (*trans*-resveratrol and ε-viniferin) analyzed in the present work, so they are not equally affected by the same factors.

Finally, it is noteworthy that the compounds corresponding to each cluster are related by its chemical structure. This could mean that the influence of the environmental factors such as light, temperature and precipitations have different impact on the polyphenols depending on the family of the specific compound. Although the comparison of the results obtained in the present study with other works is very difficult as each study consider distinct factors and use different protocols, other authors also found different influence of environmental factors on the synthesis and accumulation of the different families of phenolic compounds. Therefore, Blancquaert et al. [40] studied the evolution of flavonoids under altered temperature and light conditions of grapes during ripening in two different vintages. Although they studied the evolution of flavonoids in grape and not in grape stem, they also found different behavior between anthocyanins and flavonols. They concluded that the reduction of UV-B light significantly decreases the amount of flavonol biosynthesis, while this parameter has low influence in the case of anthocyanins, being both light and temperature the main factors that affect their synthesis and accumulation. Teixeira et al. [34] have also concluded that there exists different behavior between anthocyanins and proanthocyanidins with temperature and radiation, and identified flavan-3-ol compounds and proanthocyanidins as the most stable phenolics under diverse growing conditions.

## 4. Conclusions

The results showed that there exist significant differences in antioxidant activity, TPC and TF among grape stem from different varieties, and also between stems from the same grape variety but different vintages. The year that presented the highest number of days with extreme temperature conditions and accumulated precipitations during the time of grape development, veraison and ripeness (2016), was also the year in which the grape stems presented the lowest TPC, TF, antioxidant activity and concentration values of individual phenolic compounds. Regarding individual phenolic compounds, in general, Mazuelo presented higher concentration values of the different phenolic compounds than Garnacha and Tempranillo. It was also found that climatic conditions during the specified period of time had different impact on the synthesis of polyphenols depending on the family of the specific compound. Specifically, quercetin and its derivative, catechin, procyanidin B1 and gallic acid were the compounds most affected from one vintage to another, while the stilbenes presented a marked varietal component, since there has hardly been variation in their concentration from one year to another in any of the varieties studied in comparison with the rest of phenolic compounds analyzed.

In view of these results, it is important to consider that not only the variety can have a deep impact on the phenolic composition of a grape stem extract. Changes in the phenolic composition within the same variety among different vintages could significantly determine the future application of their corresponding extract. Therefore, the knowledge of the composition of grape stem extracts of different grape varieties and vintages is very important, as offers the possibility to select the most suitable application for each extract. 

## Figures and Tables

**Table 1:** Calibration parameters for each individual phenolic compound.

Phenolic Compound	λ (nm)	Concentration Range (µg/mL)	Rt (min)	R^2^	LLOD (µg/mL)	LLOQ (µg/mL)
Gallic Acid	272	5.1–168.7	6.5 ± 0.2	>0.999	1.1	5.1
Ellagic Acid	367	0.5–36.3	28.7 ± 0.4	>0.999	0.4	0.5
Caftaric Acid	329	0.5–36.5	15.4 ± 0.3	>0.998	0.8	0.5
Catechin	279	40.0–133.2	17.4 ± 0.4	>0.991	1.4	40.0
*trans-*Resveratrol	306	0.5–42.6	38.8 ± 0.3	>0.997	0.9	0.5
ε-Viniferin	324	2.1–21.1	44.6 ± 0.1	>0.993	0.5	2.1
Quercetin	369	0.4–17.6	42.2 ± 0.2	>0.994	0.8	0.4
Qurecetin-3-derivative	354	7.8–78.2	30.0 ± 0.4	>0.995	2.1	7.8
Procyanidin B1	279	6.8–136.5	14.7 ± 0.3	>0.997	6.3	6.8
Malvidin-3-glucoside	526	0.4–34.6	23.3 ± 0.1	>0.998	1.1	0.4

**Table 2:** Antioxidant activity, total phenolic content (TPC) and total flavonoid content (TF) of grape stem extracts from different varieties and from two different vintages. (Mean ± SD).

Variety	Antioxidant Activity ^1^			Total Polyphenol Content ^2^	Total Flavonoid Content ^3^
	ABTS	FRAP	DPPH		
**Vintage 2016**					
GAR	0.33 ± 0.05 ^a^	0.16 ± 0.02 ^a^	0.24 ± 0.02 ^a^	34 ± 5 ^a^	1.5 ± 0.2 ^a^
MAZ	0.80 ± 0.05 ^b^	0.36 ± 0.03 ^b^	0.48 ± 0.03 ^b^	82 ± 1 ^b^	2.5 ± 0.1 ^b^
TEM	0.81 ± 0.05 ^b^	0.46 ± 0.03 ^c^	0.60 ± 0.01 ^c^	87 ± 3 ^b^	2.2 ± 0.1 ^b^
GRA	0.92 ± 0.06 ^b^	0.42 ± 0.03 ^bc^	0.58 ± 0.02 ^c^	90 ± 5 ^b^	2.2 ± 0.1 ^b^
**Vintage 2018**					
GAR	0.84 ± 0.01 ^a^	0.44 ± 0.02 ^a^	0.54 ± 0.01 ^a^	86 ± 1 ^a^	2.3 ± 0.1 ^b^
MAZ	1.51 ± 0.17 ^c^	0.80 ± 0.09 ^b^	0.81 ± 0.08 ^b^	172 ± 6 ^c^	2.8 ± 0.1 ^c^
TEM	1.12 ± 0.03 ^b^	0.57 ± 0.04 ^a^	0.63 ± 0.01 ^a^	135 ± 7 ^b^	3.0 ± 0.1 ^d^
CHA	0.78 ± 0.02 ^a^	0.43 ± 0.02 ^a^	0.57 ± 0.02 ^a^	84 ± 2 ^a^	2.0 ± 0.1 ^a^
CS	0.92 ± 0.05 ^ab^	0.50 ± 0.02 ^a^	0.64 ± 0.03 ^a^	95 ± 4 ^a^	1.9 ± 0.1 ^a^

^1^ expressed as mmol Trolox/g extract; ^2^ expressed as mg gallic acid/g extract; ^3^ expressed as mg quercetin/g extract. GRA: Graciano, MAZ: Mazuelo, TEM: Tempranillo, GAR: Garnacha, CHA: Chardonnay and CS: Cabernet Sauvignon. Different letters in the same column and within the same vintage indicate significantly different results among varieties (ANOVA, significance level: 0.05). In all cases, *n* = 6.

**Table 3:** Phenolic composition (mg/g extract) of the different grape stem extracts analyzed in this study (mean ± SD).

	Gallic Acid	Ellagic Acid	Caftaric Acid	Catechin	*trans-*Resveratrol	ε-Viniferin	Quercetin	Quercetin-3-Derivative	Procyanidin B1	Malvidin-3-Glucoside	Unknown Anthocyanin
**Vintage 2016**											
GAR	0.12 ± 0.03 ^a^	0.031 ± 0.007 ^a^	0.05 ± 0.01 ^a^	1.0 ± 0.2 ^a^	0.06 ± 0.01 ^a^	0.33 ± 0.05 ^ab^	0.014 ± 0.002 ^a^	0.24 ± 0.06 ^a^	0.4 ± 0.1 ^a^	0.03 ± 0.01 ^a^	<LD ^a^
MAZ	0.23 ± 0.01 ^b^	0.028 ± 0.001 ^a^	0.13 ± 0.00 ^b^	1.2 ± 0.1 ^ab^	0.24 ± 0.02 ^b^	0.69 ± 0.03 ^c^	0.043 ± 0.002 ^c^	0.99 ± 0.03 ^d^	0.4 ± 0.0 ^a^	0.09 ± 0.00 a	0.10 ± 0.00 ^b^
TEM	0.14 ± 0.02 ^a^	0.043 ± 0.009 ^a^	0.12 ± 0.01 ^b^	1.0 ± 0.2 ^a^	0.04 ± 0.01 ^a^	0.20 ± 0.06 ^a^	0.029 ± 0.008 ^b^	0.70 ± 0.06 ^c^	0.5 ± 0.1 ^a^	0.48 ± 0.04 ^b^	0.22 ± 0.02 ^c^
GRA	0.18 ± 0.00 ^ab^	0.042 ± 0.002 ^a^	0.14 ± 0.01 ^b^	1.6 ± 0.0 ^b^	0.37 ± 0.03 ^c^	0.45 ± 0.03 ^b^	0.013 ± 0.001 ^a^	0.52 ± 0.02 ^b^	1.1 ± 0.1 ^b^	0.61 ± 0.03 ^c^	0.37 ± 0.03 ^d^
**Vintage 2018**											
GAR	1.29 ± 0.06 ^c^	0.097 ± 0.004 ^a^	0.14 ± 0.01 ^a^	1.3 ± 0.1 ^a^	0.13 ± 0.01 ^c^	0.46 ± 0.04 ^b^	0.073 ± 0.003 ^b^	0.62 ± 0.03 ^ab^	0.9 ± 0.1 ^a^	0.12 ± 0.00 ^b^	0.03 ± 0.00 ^a^
MAZ	1.18 ± 0.09 ^c^	0.054 ± 0.005 ^a^	0.33 ± 0.03 ^b^	3.5 ± 0.3 ^b^	0.30 ± 0.04 ^d^	0.55 ± 0.08 ^b^	0.108 ± 0.011 ^c^	1.50 ± 0.14 ^c^	2.5 ± 0.2 ^c^	0.22 ± 0.04 ^b^	0.24 ± 0.04 ^b^
TEM	0.52 ± 0.04 ^b^	0.060 ± 0.004 ^a^	0.23 ± 0.02 ^ab^	0.9 ± 0.1 ^a^	0.04 ± 0.00 ^ab^	0.15 ± 0.03 ^a^	0.056 ± 0.004 ^b^	0.79 ± 0.05 ^b^	0.4 ± 0.0 ^a^	0.80 ± 0.06 ^c^	0.55 ± 0.07 ^c^
CHA	0.15 ± 0.02 ^a^	0.049 ± 0.006 ^a^	0.20 ± 0.02 ^a^	1.0 ± 0.2 ^a^	0.07 ± 0.01 ^b^	0.42 ± 0.08 ^b^	0.013 ± 0.001 ^a^	0.39 ± 0.05 ^a^	0.7 ± 0.1 ^a^	nd ^a^	nd ^a^
CS	0.25 ± 0.04 ^a^	0.089 ± 0.015 ^a^	0.26 ± 0.03 ^ab^	2.6 ± 0.6 ^b^	0.01 ± 0.00 ^a^	0.28 ± 0.06 ^ab^	0.013 ±0.002 ^a^	0.75 ± 0.07 ^ab^	1.8 ± 0.4 ^b^	0.07 ± 0.01 ^ab^	0.01 ± 0.00 ^a^

Results expressed as mg/g extract (*n* = 9). GRA: Graciano, MAZ: Mazuelo, TEM: Tempranillo, GAR: Garnacha, CHA: Chardonnay and CS: Cabernet Sauvignon. Different letters in the same column and within the same vintage, indicate significantly different results among varieties (ANOVA, significance level: 0.05). nd, not detected.

## Data Availability

The data presented in this study are available on request from the corresponding author. The data are not publicly available due to data protection and privacy policy of some collaborating entities in the research project that funded this work.

## Supplementary Materials

The following are available online at https://www.mdpi.com/article/10.3390/biom11081221/s1, Figure S1: Inferential analysis of different spectrophotometric parameters (ABTS, DPPH, FRAP, TPC and TF) of grape stems from three different varieties (GAR = Garnacha, MAZ = Mazuelo and TEM = Tempranillo) and two vintages (2016 and 2018), Figure S2: Inferential analysis of phenolic compounds identified and quantified in different grape stem extracts from 2016 vintage (GAR = Garnacha; MAZ = Mazuelo; TEM = Tempranillo; GRA = Graciano), Figure S3: Inferential analysis of phenolic compounds identified and quantified in different grape stem extracts from 2018 vintage (GAR = Garnacha; MAZ = Mazuelo; TEM = Tempranillo; GRA = Graciano), Figure S4: Inferential analysis phenolic compounds identified in grape stems from three different varieties (GAR = Garnacha, MAZ = Mazuelo and TEM = Tempranillo) and two vintages (2016 and 2018).
