# Supplementary material for: Phenolic Composition of Grape Stems from Different Spanish Varieties and Vintages
Source: Biomolecules. 2021 Aug 16;11(8):1221. doi: 10.3390/biom11081221 (PMC8392641; doi:10.3390/biom11081221)
Supplement: Supplementary file 1 [file biomolecules-11-01221-s001.zip › biomolecules-1330285-supplementary.pdf]

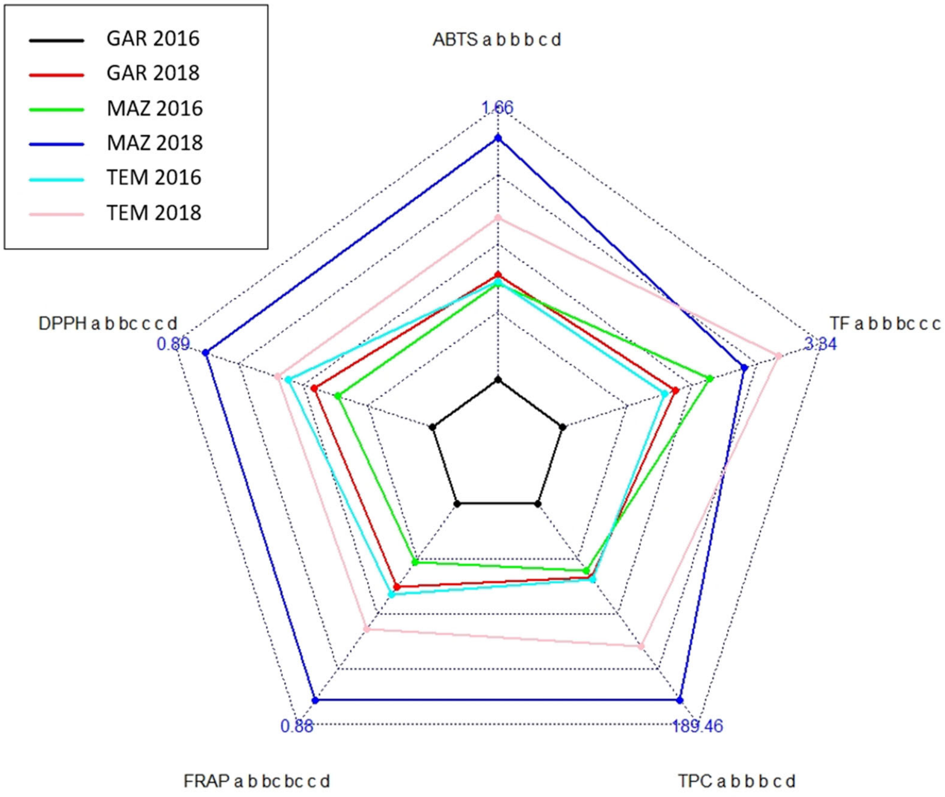

Figure S1: Inferential analysis of different spectrophotometric parameters (ABTS, DPPH, FRAP, TPC and TF) of grape stems from three different varieties (GAR=Garnacha, MAZ=Mazuelo and TEM=Tempranillo) and two vintages (2016 and 2018). Numbers in blue color represent the maximum value (expressed as mmol Trolox/g extract for the ABTS, DPPH and FRAP measurements, as mg gallic acid/g extract for TPC and as mg wuercetin/g extract for TF) in the scale of concentrations for each compound. Different letters in the same compound indicate significantly different concentration values among varieties ($p < 0.05$), and should be read from a to d (from lowest to highest values).

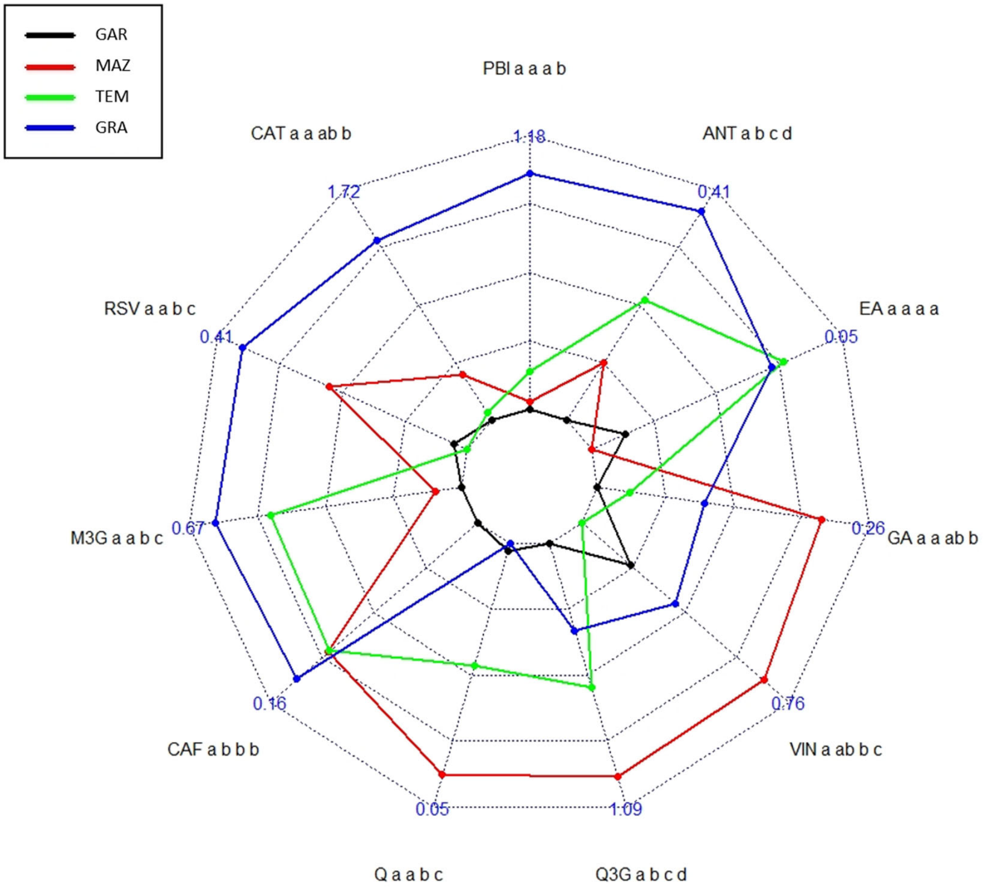

Figure S2. Inferential analysis of phenolic compounds identified and quantified in different grape stem extracts from 2016 vintage (GAR=Garnacha; MAZ=Mazuelo; TEM=Tempranillo; GRA= Graciano): CAT (catechin), PB1 (procyanidin B1); ANT (unknown anthocyanin); EA (ellagic acid); GA (gallic acid); VIN (ε-viniferin); Q3G (quercetin-3-derivative); Q (quercetin); CAF (caftaric acid); M3G (malvidin-3-glucoside); RSV (trans-resveratrol). Numbers in blue color represent the maximum value (expressed in mg/g extract) in the scale of concentration for each compound. Different letters in the same compound indicate significantly different concentration values among varieties ($p < 0.05$), and should be read from a to d (from lowest to highest values)

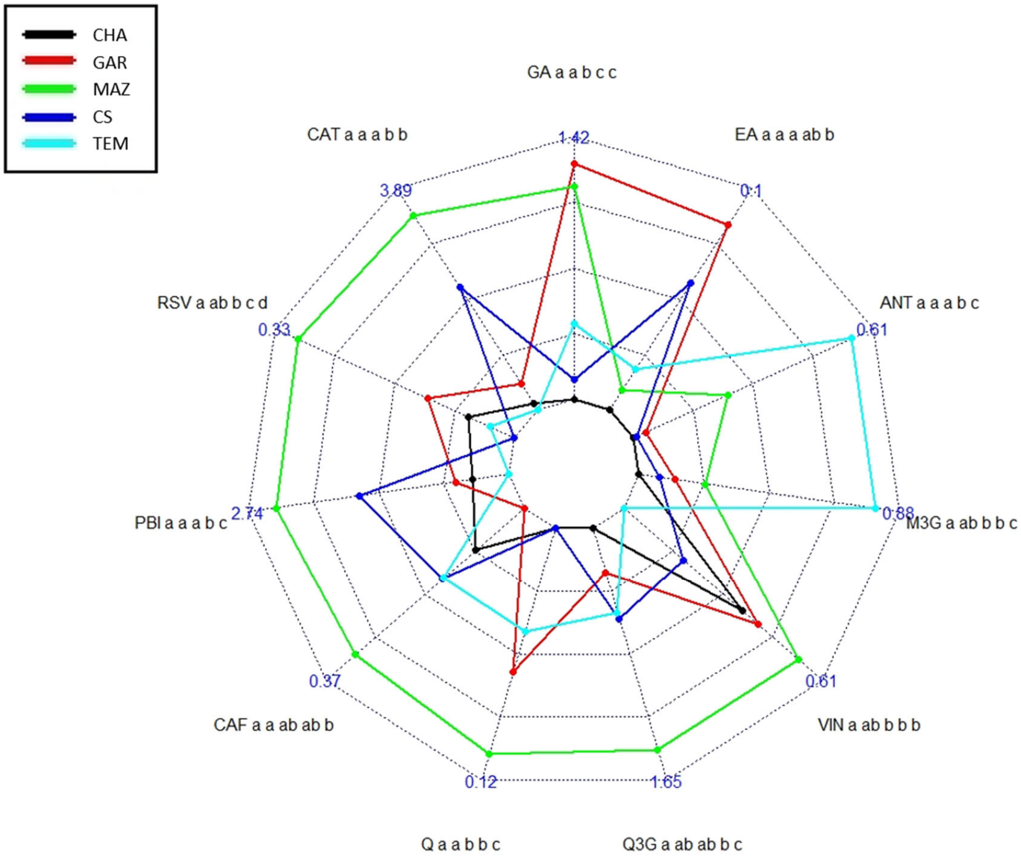

Figure S3. Inferential analysis of phenolic compounds identified and quantified in different grape stem extracts from 2018 vintage (CHA=Chardonnay; GAR=Garnacha; MAZ=Mazuelo; CS=Cabernet Sauvignon; TEM=Tempranillo): CAT (catechin), PB1 (procyanidin B1); ANT (unknown anthocyanin); EA (ellagic acid); GA (gallic acid); VIN (ε-viniferin); Q3G (quercetin-3-derivative); Q (quercetin); CAF (caftaric acid); M3G (malvidin-3-glucoside); RSV (*trans*-resveratrol). Numbers in blue color represent the maximum value (expressed in mg/g extract) in the scale of concentrations for each compound. Different letters in the same compound indicate significantly different concentration values among varieties ($p < 0.05$), and should be read from a to d (from lowest to highest values).

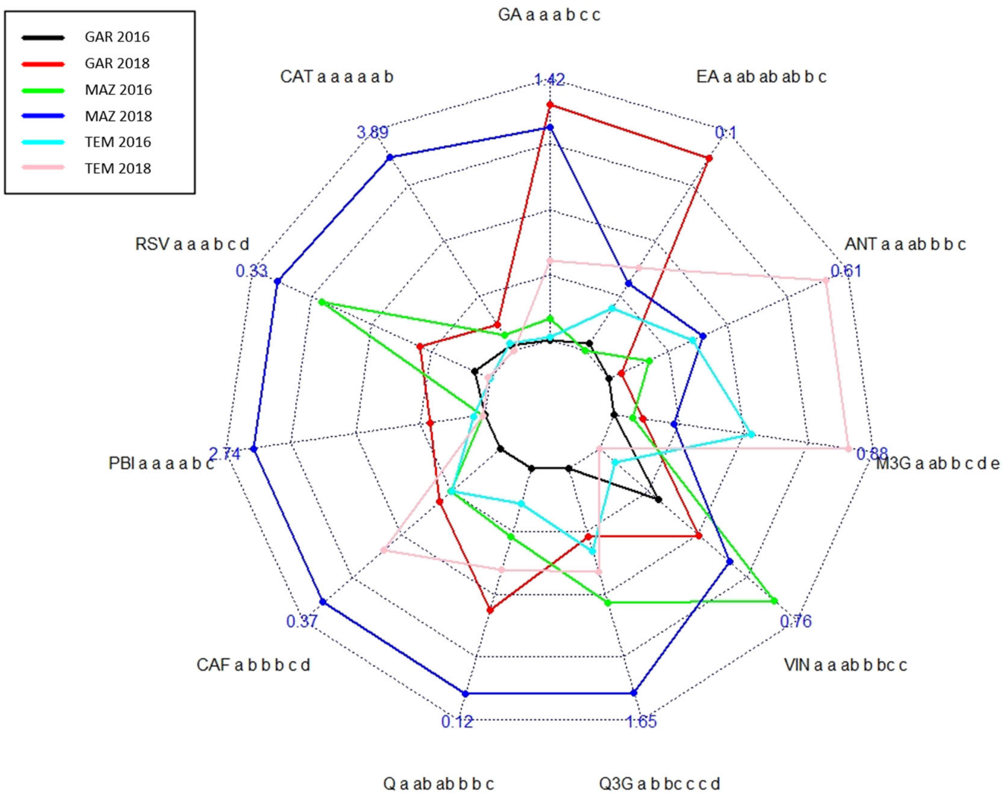

Figure S4: Inferential analysis of phenolic compounds identified in grape stems from three different varieties (GAR=Garnacha, MAZ=Mazuelo, TEM=Tempranillo) and two vintages (2016 and 2018): CAT (catechin), PB1(procyanidin B1); ANT (unknown anthocyanin); EA (ellagic acid); GA (gallic acid); VIN (ε-viniferin); Q3G (quercetin-3-derivative); Q (quercetin); CAF (caftaric acid); M3G (malvidin-3-glucoside); RSV (*trans*-resveratrol). Numbers in blue color represent the maximum value (expressed in mg/g extract) in the scale of concentrations for each compound. Different letters in the same compound indicate significantly different concentration values among varieties ($p < 0.05$), and should be read from a to d (from lowest to highest values).

Fresh Garnacha grape stem (2018)

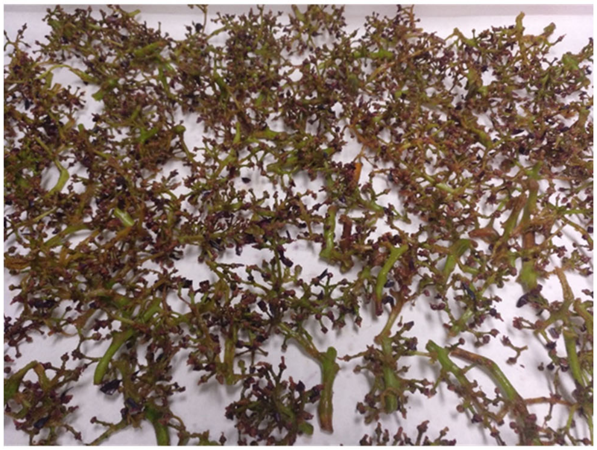

Fresh Mazuelo grape stem (2018)

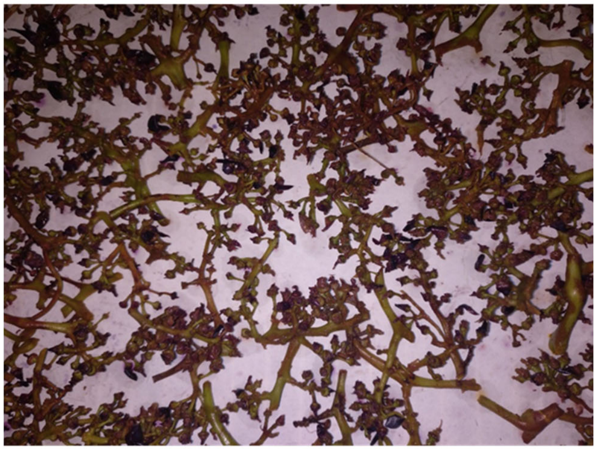

Fresh Tempranillo grape stem (2018)

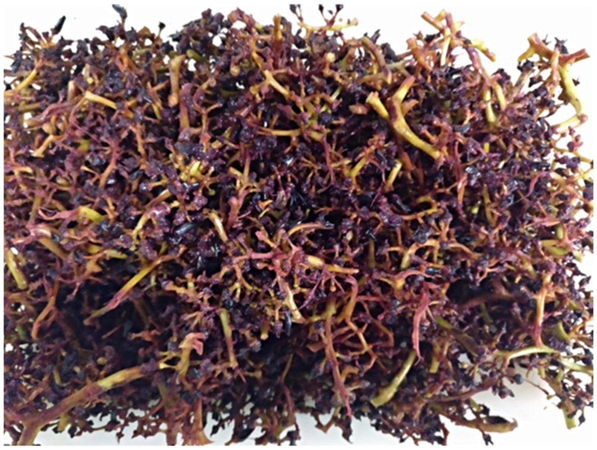

Figure S5: Fresh grape stems from 2018 vintage
